# A Ready-to-Use Single- and Duplex-TaqMan-qPCR Assay to Detect and Quantify the Biocontrol Agents *Trichoderma asperellum* and *Trichoderma gamsii*

**DOI:** 10.3389/fmicb.2018.02073

**Published:** 2018-08-31

**Authors:** Donato Gerin, Stefania Pollastro, Celeste Raguseo, Rita M. De Miccolis Angelini, Francesco Faretra

**Affiliations:** ^1^Department of Soil, Plant and Food Sciences, University of Bari Aldo Moro, Bari, Italy; ^2^Public Laboratory of Research SELGE Network No. 14, Bari, Italy

**Keywords:** biocontrol agents, esca, grapevine, soil, probe, real time PCR

## Abstract

*Trichoderma asperellum* strain icc012 and *Trichoderma gamsii* strain icc080, the microbial active ingredients of Remedier^TM^ (ISAGRO, Novara, Italy), are biocontrol agents (BCAs) employable for crop protection against a wide range of fungal pathogens, including soil-borne pathogens and fungi involved in grapevine trunk disease. In this study, single and duplex real-time quantitative PCR (qPCR) methods to detect and quantify *T. asperellum* and *T. gamsii* were developed. Primers/probe sets were designed on the *T. asperellum* and *T. gamsii rpb2* genes and tested for specificity on a panel of microorganisms commonly associated with grape wood and soil. No differences were observed comparing single- and duplex-qPCR assays on different BCAs, 1 pg of target DNA was detected approximately at *C*_q_ = 34. *R*^2^-values and the efficiency were always equal to 0.99 and >80%, respectively. The detection limit of the duplex-qPCR assay on artificially inoculated samples was 2 × 10^3^ and 4 × 10^4^ conidia g^-1^ of grape wood tissue and soil, respectively. The methods will be useful to better schedule BCA application in the field and in grapevine nurseries, as well as for investigating the dynamic of BCA populations.

## Introduction

The application of biocontrol agents (BCAs) in sustainable agriculture model represents an eco-friendly strategy compared with the use of synthetic plant protection products (PPPs) for managing weeds, insects and fungal pathogens including fungicide-resistant mutants (Jensen et al., 2016; Bruce et al., 2017; Rotolo et al., 2018).

The genus *Trichoderma*, a cosmopolitan inhabitant of soil and plant root ecological niches includes the most explored BCA species, representing over 60% of all the currently registered BCAs used for the management of plant pathogens (Benítez et al., 2004; Harman et al., 2004; Mukherjee et al., 2013; Hyder et al., 2017; Sharma et al., 2017). Their biological activity is closely related to the ability of: (i) producing a wide range of lysing enzymes; (ii) degrading substrates; (iii) possessing high resistance to microbial inhibitors; (iv) competing for nutrients and space, (v) acting directly through mycoparasitism, (vi) producing antifungal metabolites; (vii) inducing systemic resistance in plants (Strange, 1993; Harman et al., 2004; Shoresh et al., 2005; Reino et al., 2008; Lorito et al., 2010; Qualhato et al., 2013). *Trichoderma* spp. are fast-growing, strong spore producers and stimulate plant growth through the production of promoting molecules (e.g., Eziashi et al., 2007; Vinale et al., 2008; Hermosa et al., 2012; Singh et al., 2014).

Since combining two or more beneficial microbes in a biopesticide would be advantageous to BCA management (Raupach and Kloepper, 1998), the mixture of *T. asperellum* strain icc012 and *T. gamsii* strain icc080 is used in Remedier^TM^ to increase the activity and widening the environmental adaptability (Liguori, 2016). This microbial pesticide is registered against soil-borne pathogens affecting horticultural crops and turfs, and it is the only BCA-based PPP allowed in Italy to control pathogens associated with grapevine trunk diseases (GTDs).

To date, 133 fungal species belonging to 34 genera have been associated with GTDs affecting, singularly or simultaneously, table and wine grapes as well as rootstocks. PPPs effective in controlling GTDs-associated fungi are still lacking, and the BCAs *Trichoderma atroviride* and *Trichoderma harzianum* were the ones most studied for their effectiveness (Gramaje et al., 2018). On the other hand, information on *T. asperellum* and *T. gamsii* refer mostly to their use for cutting wounds protection while the population dynamics has been scarcely studied and appropriate monitoring systems are lacking. Yet, the monitoring of these BCAs in natural environments is essential to evaluate their effectiveness and scheduling their applications (Torsvik and Øvreås, 2002).

Molecular detection and quantification of fungal species (Filion et al., 2003; Lievens et al., 2006; López-Mondéjar et al., 2010; Sharma and Salwan, 2017) are substituting for conventional techniques, such as those based on the assessment of colony forming units (CFU) and on chemical, biological and immunological assays (Thornton et al., 1994). In fact, the differentiation of *Trichoderma* using morphological characteristics is very difficult, due to the scarcity of specific traits (Błaszczyk et al., 2011; Devi et al., 2012). Different qPCR and qRT-PCR assays have therefore been proposed for the quantification of *T. harzianum* (Rubio et al., 2005; López-Mondéjar et al., 2010; Beaulieu et al., 2011), *T. atroviride* (Cordier et al., 2007; Savazzini et al., 2008) and *Trichoderma* spp. (Hagn et al., 2007; Kim and Knudsen, 2008).

This study aimed at developing a molecular qPCR tool for an easy detection and quantification of *T. asperellum* strain icc012 and *T. gamsii* strain icc080. Comparisons between single- and duplex-qPCR assays were performed, then the assays were validated on fungal DNA extracts from grapevine wood tissue and soil samples contaminated with different concentrations of BCAs conidia.

## Materials and Methods

### Strains and Media

*Trichoderma asperellum* icc012 and *T. gamsii* icc080 were kindly supplied by Isagro SpA (Novara, Italy). Non-target species of fungi, yeasts and bacteria used were from the microbial culture collection of our Department.

Fungi and yeasts were routinely grown on potato dextrose agar (PDA: infusion from 200 g peeled and sliced potatoes kept at 60 ± 1°C for 1 h, 20 g dextrose per liter of distilled water, pH adjusted to 6.5, and 20 g agar Oxoid No. 3) at 21 ± 1°C in the darkness. Alternatively, bacteria were routinely grown on Luria-Bertani medium (LB: 10 g tryptone-peptone, 5 g yeast extract, pH adjusted to 7.0, and 14 g agar per liter of distilled water) at 25 ± 1°C in the darkness.

### Primers/Probe Sets Design

Sequences of the genes translation elongation factor 1-alpha (*tef1*), endochitinase 42 (*ech42*) and RNA polymerase B subunit II (*rpb2*) of target and non-target *Trichoderma* species were retrieved from GenBank^1^, and aligned using the SeqMan Pro software (DNASTAR, Lasergene, Madison, WI, United States). Based on the highest presence of species-specific single-nucleotide polymorphisms (SNPs), the *rpb2* gene was selected and the sequences of different *Trichoderma* species (**Supplementary Figure S1**), including 64 sequences of *T. asperellum* and 10 sequences of *T. gamsii*, were aligned and examined *in silico* using the SeqMan Pro software (DNASTAR). The SNPs identified in *T. asperellum* and *T. gamsii* were used. The primers/probe sets (**Table 1**) were manually designed primarily in order to include the specific SNPs in the 3′ position of the primer forward (base 504 for *T. asperellum* reference sequence GenBank accession No. GU198278.1) and probe (base 806 for *T. gamsii* reference sequence GenBank accession No. KJ665270.1). Other SNPs in different positions of *T. asperellum* and *T. gamsii* primers/probe sets were also recorded. The absence of secondary structures and dimers and the feasibility of the use of Taq Man^®^-qPCR were verified using the Primer Express 3.0 software (Applied Biosystems, Foster City, CA, United States). Primers/probe sets were custom-synthesized (Macrogen, Seoul, South Korea) including FAM (6-carboxyfluorescein) and HEX (6-hexachlorofluorescein) fluorescent dyes to label the *T. asperellum* and *T. gamsii* probes, respectively.

**Table 1:** Primers/probe sets for *T. asperellum* and *T. gamsii*.

Species	Primer name	Primer/Probe sequence (5′–3′)^∗^
*T. asperellum*	Ta_rpb2_fw	GGAGGTCGTTGAGGA GTACGAA
	Ta_rpb2_rev_3	TTGCAGATAGGATTTAC GACGAGT
	Ta_rpb2_probe	FAM-CGCTGAGGTATCCCCAT GCGACA-BHQ1
*T. gamsii*	Tg_rpb2_fw	GCCACCTGGTTTT GACCAAGGA
	Tg_rpb2_rev	CGCACCAGCCCTGATCA
	Tg_rpb2_probe	HEX-CCTCCAGAAGACCCAAGC ATGAAGCTC-BHQ1

*^∗^Underlined letters correspond to the specificity at the 3′ end.*

### DNA Extraction From Trichoderma and qPCR Conditions

Genomic DNA of both BCAs and non-target fungi and yeasts was extracted from 5-day-old colonies grown at 21 ± 1°C on cellophane disks overlaid on PDA, according to the protocol of De Miccolis Angelini et al. (2010). DNA from bacteria was extracted according to Rotolo et al. (2016). Quantity and quality of DNA was assessed using a NanoDrop 2000 Spectrophotometer (Thermo Fisher Scientific, Waltham, MA, United States).

Amplifications were performed in a CFX96^TM^ Real-Time PCR Detection System Thermal Cycler (Bio-Rad Laboratories, Hercules, CA, United States) whereas the CFX Manager^TM^ version 1.0 software (Bio-Rad Laboratories, Hercules, CA, United States) was used for experimental setup and data analysis.

PCR mixes consisted of 6.25 μL of Sso Advanced^TM^ Universal Probes Supermix (Bio-Rad Laboratories, Hercules, CA, United States), 250 nM of each primer, 150 nM of each probe, 1 (single-qPCR) or 2 (duplex-qPCR) μL of DNA template, and ultrapure water to 12.5 μL. Thermal cycling conditions were 95°C for 2 min, followed by 40 cycles of 95°C for 5 s and 64.5°C for 30 s. All qPCR assays were run with appropriate controls, including the non-template control (NTC). Two replicates of each sample were analyzed, and reactions were repeated at least twice.

The qPCR products were loaded on 1.5% (w/v) agarose gel (Bio-Rad Laboratories, Hercules, CA, United States), including GelRed (Società Italiana Chimici, Rome, Italy), electrophoresed for 110 min at 110 V, and visualized with a Bio-Rad Gel Doc XR 2.0 system (Bio-Rad Laboratories, Hercules, CA, United States).

### Specificity and Sensitivity Assays

The specificity of the TaqMan-based duplex-qPCR was assessed on a panel of microorganisms commonly associated with grapevines (**Table 2**). Genomic grapevine and soil DNAs were also used as external negative control to exclude cross-reaction of the primers/probe sets, and all qPCR assays were run with appropriate controls, including the NTCs.

**Table 2:** Quantification cycle (C_q_) values of *T. asperellum* (TA) and *T. gamsii* (TG) primers/probe sets tested in the specificity assay through duplex-qPCR.

Species	Host	Geographic origin	C_q_ (TA/TG)^∗^
**Target Species**			
*Trichoderma asperellum* (icc 012)	Unknown	Unknown	21.3/-
*Trichoderma asperellum* (TA1)	Nasturtium	Terlizzi, Bari, Italy	21.4/-
*Trichoderma asperellum* (B6)	Unknown	Unknown	20.5/-
*Trichoderma asperellum* (CBS 121698)	Houhere	New Zealand	21.58/-
*Trichoderma asperellum* (CBS 123775)	Soil	South Africa	20.95/-
*Trichoderma asperellum* (CBS 125558)	Soil	Georgia, United States	21.14/–
*Trichoderma gamsii* (icc 080)	Unknown	Unknown	–/22.8
*Trichoderma gamsiii* (A8)	Unknown	Unknown	–/21.3
*Trichoderma gamsii* (CBS 120074)	Soil	Sardinia, Italy	–/21.79
*Trichoderma gamsii* (CBS 120075)	Soil	Sardinia, Italy	–/22.73
*Trichoderma gamsii* (CBS 120961)	Soil	Turkey	–/22.84
*Trichoderma gamsii* (CBS 123300)	Eucalyptus	Australia	–/23.57
**Fungal And Yeast Non-Target Species**			
*Alternaria alternata*	Unknown	Rutigliano, Apulia, Italy	–/–
*Armillaria mellea*	Peach	Mottola, Apulia, Italy	–/–
*Aspergillus niger* (AN1)	Grape	Sava, Apulia, Italy	–/–
*Aureobasidium pullulans*	Grape	Unknown	–/–
*Botrytis cinerea* (SAS56)	Monoascopore	From a sexual cross	–/–
*Cylindrocarpon destructans* (Cy37)	Peach	Policoro, Basilicata, Italy	–/–
*Cylindrocarpon destructans* (Cy38)	Peach	Policoro, Basilicata, Italy	–/–
*Cylindrocarpon liriodendri*	Peach	Policoro, Basilicata, Italy	–/–
*Fusarium oxysporium* (IV48)	Grape	Foggia, Apulia, Italy	–/–
*Fusarium* sp. (IV100)	Grape	Tralbaya, Libanon	–/–
*Fusarium* sp. (IV17)	Wheat	Unknown	–/–
*Fusarium* sp. (IV54)	Grape	Foggia, Apulia, Italy	–/–
*Fusarium solani* (IV105)	Peach	Unknown	–/–
*Gliocladium roseum* (IV101)	Palm	Unknown	–/–
*Monilia laxa*	Cherry	Turi, Apulia, Italy	–/–
*Neofusicoccum vitifusiforme*	Grape	Rutigliano, Apulia, Italy	–/–
*Penicilliumexpansum*	Grape	Unknown	–/–
*Phaeomoniella chlamydospora*	Grape	Ginosa, Apulia, Italy	–/–
*Phomopsis viticola*	Grape	Rutigliano, Apulia, Italy	–/–
*Pythium litorale*	Peach	Unknown	–/–
*Rhizoctonia solani*	Carnation	Unknown	–/–
*Rosellinia necatrix*	Grape	Ortona, Abruzzo, Italy	–/–
*Sclerotinia sclerotiorum*	Melon	Taranto, Apulia, Italy	–/–
*Trichoderma aggressivum f. europeaum*	Unknown	Unknown	–/–
*Trichoderma atroviride* (FV54)	Unknown	Unknown	–/-
*Trichoderma atroviride* (FV271)	Unknown	Unknown	–/–
*Trichoderma atroviride* (P1)	Unknown	Unknown	–/–
*Trichoderma crassum* (CBS 336.93)	Soil	Québec, Canada	–/–
*Trichoderma effusum* (DAOM 230007)	Unknown	Unknown	–/–
*Trichoderma erinaceum* (CBS 124604)	Cacao	Perù	–/–
*Trichoderma erinaceum* (CBS 117088)	Soil	Ko Lan, Thailand	–/–
*Trichoderma harzianum*	Nasturtium	Terlizzi, Apulia, Italy	–/–
*Trichoderma harzianum*	Nasturtium	Terlizzi, Apulia, Italy	–/–
*Trichoderma harzianum* (FV146)	Unknown	Unknown	–/–
*Trichoderma harzianum* (FV178)	Unknown	Unknown	–/–
*Trichoderma harzianum* (T22)	Unknown	Unknown	–/–
*Trichoderma harzianum* (T34)	Unknown	Unknown	–/–
*Trichoderma harzianum* (Tch8)	Unknown	Unknown	–/–
*Trichoderma harzianum* (FV185)	Unknown	Unknown	–/–
*Trichoderma hirsutum* (Cas-1)	Unknown	Unknown	–/–
*Trichoderma koningii* (CBS 457.96)	Soil	North Holland, Netherlands	–/–
*Trichoderma koningii* (CBS 458.96)	Soil	North Holland, Netherlands	–/–
*Trichoderma koningiopsis* (CBS 132570)	Bamboo	Aquitaine, France	–/–
*Trichoderma koningiopsis* (Tch5)	Unknown	Unknown	–/–
*Trichoderma longibrachiatum* (MK1)	Unknown	Unknown	–/–
*Trichoderma minutisporum* (CBS 341.93)	Soil	Québec, Canada	–/–
*Trichoderma oblongisporum* (CBS 343.93)	Western red cedar	British Columbia, Canada	–/–
*Trichoderma paraviridescens*. (Tch1)	Unknown	Unknown	–/–
*Trichoderma polysporum* (CBS 337.93)	Soil	Québec, Canada	–/–
*Trichoderma polysporum* (Montr-2)	Unknown	Unknown	–/–
*Trichoderma pseudokoningii* (FV144)	Unknown	Unknown	–/–
*Trichoderma rossicum* (DAOM 230011)	Unknown	Unknown	–/–
*Trichoderma* sp. (Tch2)	Unknown	Unknown	–/–
*Trichoderma* sp. (Tch4)	Unknown	Unknown	–/–
*Trichoderma* sp. (Tch6)	Unknown	Unknown	–/–
*Trichoderma* sp. (Tch7)	Unknown	Unknown	–/–
*Trichoderma spirale* (Tch3)	Unknown	Unknown	–/–
*Trichoderma reesei*	Nasturtium	Terlizzi, Apulia, Italy	–/–
*Trichoderma virens* (CBS 116947)	Soil	Pisa, Tuscany, Italy	–/–
*Trichoderma viride* (Tch9)	Unknown	Unknown	–/–
*Verticillium dahliae*	Artichoke	Metaponto, Basilicata, Italy	–/–
**Bacterial Non-Target Species**			
*Bacillus subtilis*	Grape	Unknown	–/–
*Bacillus amyloliquefaciens*	Grape	Unknown	–/–
*Pantoea agglomerns*	Grape	Unknown	–/–
*Pseudomonas fluorescens*	Grape	Unknown	–/–
*Pseudomonas putida*	Grape	Unknown	–/–

*^∗^C_*q*_ values are the mean values of two technical replicates.*

Ten-fold serial dilutions (100 ng to 0.01 pg) of genomic DNA of *T. asperellum* icc012 and *T. gamsii* icc080 were used in sensitivity assays. The standard curves for each BCA were generated in both single- and duplex-qPCR by plotting the quantification cycle (C_q_) values vs. the Log_10_ of 10-fold serial dilutions of DNA. Comparison between single- and duplex-qPCR was done for each species. Two replicates of each dilution were analyzed, and reactions were repeated at least twice. qPCR reactions were positive if *C*_q_ value was ≤35.

### Preparation of Grape Wood and Soil Samples

Conidia of *T. asperellum* and *T. gamsii* were scraped from the surface of 7-days-old colonies grown on PDA at 25 ± 1°C in the dark and suspended in sterile distilled water containing 0.05% Tween 20. Mycelial fragments were removed through Miracloth (Calbiochem, Darmstadt, Germany). Aliquots (1 mL) of diluted conidial suspension (from 10^8^ to 10^0^ conidia mL^-1^) were used to infest 50 mg of grape wood chips (protocol 1) and 50 or 250 mg of clay soil (protocol 1 and protocol 2, respectively) previously sterilized, then ground and sieved at 2 mm to separate the gravel fraction. Samples were centrifuged (Eppendorf, Hamburg, Germany) at 14,000 rpm for 30 min and the pellet was subjected to DNA extraction. Five wood and soil samples artificially infested with *T. asperellum* or/and *T. gamsii* conidia were analyzed by qPCR and in the meantime, samples of *T. asperellum-* or *T. gamsii*-infested soil were placed on PDA and CFU were counted.

### DNA Extraction and Purification, qPCR From Wood and Soil Samples

DNA extraction from wood chips and clay soil (protocol 1) was done using the CTAB method (Cullen et al., 2001), slightly modified as described below. Samples were homogenized in 600 μL extraction buffer (0.12 M Na_2_HPO_4_, 1.5 M NaCl, 2% CTAB) with 0.5 g acid-washed glass beads 425–600 μm (Sigma-Aldrich, St. Louis, MO, United States) and 2 steel spheres (5 mm diam.). The suspension was strongly shaken for 5 min at 1,500 oscillations min^-1^ using a Mixer Mill (MM301, Retsch GmbH, Haan, Germany). The supernatant, collected after centrifugation at 14,000 rpm for 15 min, was transferred in a new 2 mL micro-tube. Extraction was carried out in 750 μL of chloroform. Nucleic acids were collected by centrifugation for 15 min at 14,000 rpm, precipitated with 750 μL of isopropanol at -80 ± 3°C for 30 min, and recovered by centrifugation at 14,000 rpm for 15 min. The pellet, washed with 200 μL of ethanol (70%), was suspended in 50 μL of ultrapure water.

DNA extraction from soil was carried out according to Martin-Laurent et al. (2001, protocol 2).

Wood-DNA extract was purified using Sepharose 6B (Sigma-Aldrich, St. Louis, MO, United States)-columns, while the soil-DNA extract was purified on both Sepharose 6B- and polyvinylpolypyrrolidone (PVPP) (Sigma-Aldrich, St. Louis, MO, United States) columns, prepared as reported by Rotolo et al. (2016). DNA concentration and purity were estimated as described above and DNA was stored at -80°C until use. To verify the success of DNA extraction, the extract was amplified by using the universal primers ITS5/ITS4, and the PCR mixture and conditions previously described. DNA extracted was directly amplified in single- and duplex-qPCR. Curves were generated by plotting the *C*_q_ values vs. the Log_10_ of the number of conidia added to the samples and BCAs were finally quantified as conidia per g^-1^ of grapevine wood tissue or soil.

## Results

### Primers/Probe Sets Specificity

**FIGURE 1:**
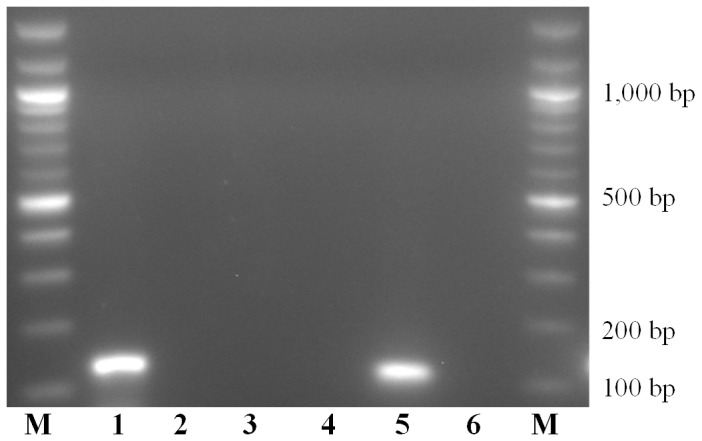
Amplicons obtained with *Trichoderma asperellum* and *T. gamsii* primers/probe sets. M: 100 bps marker; Amplification mixtures were: 1–3, *T. asperellum* primers/probe set; 4–6, *T. gamsii* primers/probe set. Samples analyzed was: 1 and 4, *T. asperellum* DNA; 2 and 5, *T. gamsii* DNA; 3 and 6, no template controls.

SNPs identified in intra- and external species alignments of the *rpb2* gene sequences were used for species-specific primers/probe sets design. The 142 and 113 bp amplicons were confirmed for *T. asperellum* and *T. gamsii*, respectively (**Figure 1**).

For duplex-qPCR assays, the best conditions to avoid unspecific amplification products were 64.5°C (annealing temperature), 250 nM/150 nM (primers/probes concentrations) and 35 cycles. The specificity of the assay was also tested against genomic DNA from 66 non-target organisms as well as from grapevines and soil. No amplicons were generated using non-target DNA from some *Trichoderma* spp. (*T. atroviride*, *T. paraviridescens*, and *T. polysporium*) not carrying the 3′ SNP in the primer forward (*T. asperellum*) or in the probe (*T. gamsii*) (**Table 2** and **Supplementary Figure S1**).

### Primers/Probe Sets Sensitivity

**FIGURE 2:**
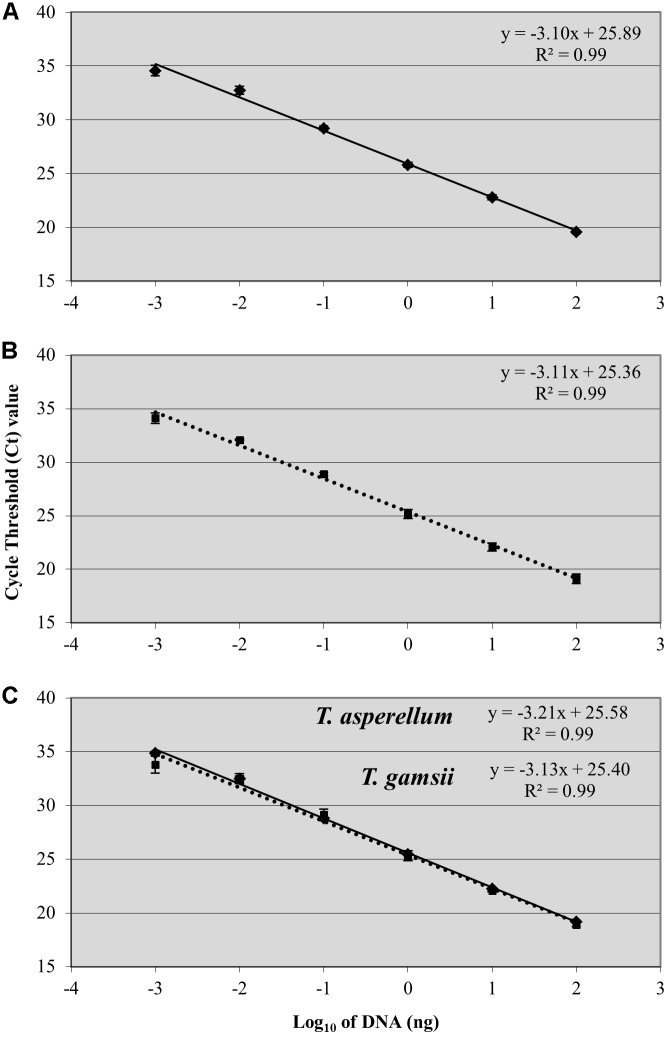
Standard curves generated by single-qPCR for *Trichoderma asperellum* **(A)** and *T. gamsii* **(B)** and duplex-qPCR **(C)**. The *C*_t_ values were plotted against the DNA concentrations expressed on a logarithmic scale. Bars mean standard error of two technical replicates.

A linear response was observed from 100 ng to 1 pg of *T. asperellum* and *T. gamsii* DNA in single-qPCR (**Figures 2A,B**). *R*^2^ and efficiency of the standard curve were always equal to 0.99 and >80%, respectively, and the linear regression slopes were -3.10 and -3.11, respectively, for *T. asperellum* and *T. gamsii*. Both *T. asperellum* and *T. gamsii* primers/probe sets showed the same sensitivity when duplexed (**Figure 2C**). In the duplex-qPCR assay, 1 pg of target DNA for both species was also detected approximately at C_q_ 34. Unspecific amplification occurred beyond the 35th cycle. In duplex-qPCR, *R*^2^ and efficiency of the standard curves were also equal to 0.99 and >80% for both primer/probe sets, while the linear regression slopes were -3.21 and -3.13 for *T. asperellum* and *T. gamsii*, respectively.

### Validation on Grapevine Wood and Soil

To validate the single- and duplex-qPCR assays, wood chips, and soil samples were artificially infested with a 10-fold dilution of conidial suspensions of *T. asperellum* and *T. gamsii* used singularly and in mixture. BCAs were only detected in all samples expected to be positive.

The detection limit in single- and duplex-qPCR was 2 × 10^3^ conidia g^-1^ of grape wood chips for both BCAs. In single-qPCR, *R*^2^ and efficiency of the standard curves were 0.99 and 131.4% for *T. asperellum* and 0.99 and 137.3% for *T. gamsii*. In the same assays, slopes values were -2.74 and -2.66 for *T. asperellum* and *T. gamsii*, respectively (**Table 3**). In duplex-qPCR, *R*^2^ and efficiency of the standard curves were 0.97 and 127.3% for *T. asperellum* and 0.99 and 137.6% for *T. gamsii*, while slope values were -2.09 for *T. asperellum* and -2.66 for *T. gamsii* (**Table 3**).

**Table 3:** Performance of the single- and duplex-qPCR assay for detection of *T. asperellum* and *T. gamsii* in grape wood and soil samples.

Target DNA (conidia added to)	Dynamic range (conidia g^-1^)	Linear regression^∗^		
		*k*	*R*^2^	*E*
**qPCR**				

**Grape wood**				
*T. asperellum*	2 × 10^8^–2 × 10^3^	-2.74	0.99	131.4%
*T. gamsii*	2 × 10^8^–2 × 10^3^	-2.66	0.99	137.3%
**Soil**				
*T. asperellum*	4 × 10^7^–4 × 10^4^	-3.75	0.99	84.7%
*T. gamsii*	4 × 10^7^–4 × 10^4^	-3.87	0.96	81.2%

**DUPLEX-qPCR**				

**Grape wood**				
*T. asperellum*	2 × 10^8^–2 × 10^3^	-2.09	0.97	127.3%
*T. gamsii*	2 × 10^8^–2 × 10^3^	-2.66	0.99	137.6%
**Soil**				
*T. asperellum*	4 × 10^7^–4 × 10^4^	-3.32	0.95	100.1%
*T. gamsii*	4 × 10^7^–4 × 10^4^	-3.92	0.97	80.0%

*Linear regression was calculated from four to five separately prepared 10-fold conidia suspension dilutions; ^∗^in the linear regression analysis: k, slope of linear regression between logarithmic values of no. of conidia and C_*q*_ values; R^*2*^, average squared regression coefficient; E, efficiency of amplification.*

Two protocols for DNA extraction from soil were preliminarily compared using different concentrations of *T. asperellum* and *T gamsii* conidia and protocol 2 worked better than protocol 1 (**Supplementary Table S1**).

In both single- and duplex-qPCR, the detection limit for *T. asperellum* and *T gamsii* was 4 × 10^4^ conidia g^-1^ of soil (**Table 3**). In single-qPCR, *R*^2^ and efficiency of the standard curves were 0.99 and 84.7% for *T. asperellum* and 0.96 and 81.2% for *T. gamsii*. In the same assays, slopes values were -3.75 and -3.87 for *T. asperellum* and *T. gamsii*, respectively (**Table 3**). On the other hand, in duplex-qPCR, *R*^2^ and efficiency of the standard curves were 0.95 and 100.1% for *T. asperellum* and 0.97 and 80.0% for *T. gamsii*. Slopes values were, respectively, -3.32 and -3.92 for *T. asperellum* and *T. gamsii*, respectively (**Table 3**).

BCAs quantification obtained by qPCR assays always agreed with the CFU formed on PDA medium.

## Discussion

A fast reliable and sensitive species-specific method for detecting and quantifying the BCAs *T. asperellum* and *T. gamsii*, that are also the bioactive ingredients of the biofungicide Remedier^TM^ (strains icc012 and icc080, respectively), used to control pathogens associated with GTDs as well as soil-borne and turf pathogens, was developed.

qPCR represents an alternative tool for an efficient quantification of individual fungal species through the estimation of DNA (Lievens and Thomma, 2005). In the current study, single- and duplex-qPCRs based on the uniqueness of SNPs identified on the single-copy gene encoding the *rpb2* were set up and standardized.

*rpb2* is a widely studied gene, whose many sequences are available in GenBank for homology comparisons. According to the *in silico* analysis, SNP in the base position 504 of *T. asperellum* and 806 of *T. gamsii* were recognized to specifically discriminate the target BCAs from the non-target fungi, including *T. harzianum* and *T. viride*, that are commonly detected in soil and currently used for crop protection (Druzhinina et al., 2011). Less specific SNPs were detected on the other two examined gene sequences corresponding to *ech42* and *tef1*. However, the latter gene proved unsuitable for our purpose, although species-specific primers based on the *tef1* gene had been proposed for the identification of *T. asperellum*, *T. longibrachiatum* and *T. virens* (Devi et al., 2012).

The specificity against fungal and bacterial species associated with different crops, with special reference to GTD pathogens, was tested *in silico* and by qPCR. The 142 and 113 bp amplicons identified for *T. asperellum* and *T. gamsii*, respectively, discriminated both the BCAs present in the biopesticide Remedier. The single-base mismatch in 3′ position of the primer forward or of the probe were sufficient for avoiding false negatives and for discrimination from the majority of other *Trichoderma* species and other fungi. Some *Trichoderma* species, which are occasionally found (Samuels et al., 2010; Braithwaite et al., 2017), did not carry the single-base mismatch in 3′ position (**Supplementary Figure S1**). In these species other SNPs present in different positions of the *T. asperellum* and *T. gamsii* primers/probe sets could allow the discrimination from the target species as observed for *T. atroviride, T. paraviridescens*, *and T. polysporium* analyzed in duplex-qPCR (**Table 2**).

Starting from DNA extracted from pure culture, the detection limit was 1 pg, in agreement with other studies using single-copy nuclear genes for qPCR (Ridgway and Stewart, 2000; Bluhm et al., 2002; Dodd et al., 2004; Fredlund et al., 2008; Scauflaire et al., 2012). According to provision for quantitative real-time PCR experiments, the linear dynamic range was extended to six different Log10 DNA amounts (Bustin et al., 2009). Slopes of the linear regression of qPCR assay ranged between -3.1 and -3.6, corresponding to a PCR efficiency of between 80 and 100%, and the *R*^2^-value was always ≥0.99.

When BCAs added to grapevines or soil samples were quantified in single- and duplex-qPCR, the *R*^2^-values were always ≥0.95, but the slope values (-2.0 to -2.7) indicated a slight decrease in the efficiency of the qPCR, caused by the presence of potential inhibitors (i.e., polyphenols, polysaccharides, humic acids, and metals) co-extracted with the DNA and inhibiting PCR reactions by decreasing the Taq polymerase activity or by limiting template’s availability (Kermekchiev et al., 2009; Opel et al., 2010). Assuming 100% DNA recovery, the protocol quantified up to 2 × 10^3^ and 4 × 10^4^ conidia g^-1^ in plants and soil, respectively. These results agree with those reported for fungi other than *Trichoderma* (Selma et al., 2008; Garrido et al., 2009) and for other *Trichoderma* species, such as *T. virens* (Dodd et al., 2004; Oskiera et al., 2017).

## Conclusion

The duplex-qPCR assay represents a useful tool for the simultaneous detection and quantification of *T. asperellum* and *T. gamsii* and can assist in learning more about fungal activity, survival and spread in large-scale monitoring. Accordingly, BCA populations can be monitored on grapevines and different crops, both in the field and nursery.

## Author Contributions

DG, SP, RDMA, and FF conceived and planned the experiments. DG and CR performed the experiments. DG and SP took the lead in writing the manuscript. SP, RDMA, and FF supervised the research. All authors contributed to the interpretation of the results, provided critical feedback and helped to shape the research, analysis, and manuscript.

## Conflict of Interest Statement

The authors declare that the research was conducted in the absence of any commercial or financial relationships that could be construed as a potential conflict of interest.

## Footnotes

1 http://www.ncbi.nlm.nih.gov/

## Supplementary Material

FIGURE S1 SNPs identification in *T. asperellum* **(A)** *and T. gamsii* **(B)** *rpb2* gene sequences for primers/probe sets design. ^∗^For each *Trichoderma* spp., GenBank accession number of representative *rpb2* gene sequences is included between parenthesis. SNPs as compared to *T. asperellum* **(A)** and *T. gamsii* **(B)** are displayed with a different color.

TABLE S1 Detection of *T. asperellum* DNA extracted with two extraction protocols by qPCR. ^∗^50 mg (protocol 1) and 250 mg (protocol 2). n.a., no amplification. Values are mean ± standard error of three biological replicates.
