# Supplementary figures and images for: A Ready-to-Use Single- and Duplex-TaqMan-qPCR Assay to Detect and Quantify the Biocontrol Agents Trichoderma asperellum and Trichoderma gamsii
Source: Front Microbiol. 2018 Aug 31;9:2073. doi: 10.3389/fmicb.2018.02073 (PMC6127317; doi:10.3389/fmicb.2018.02073)

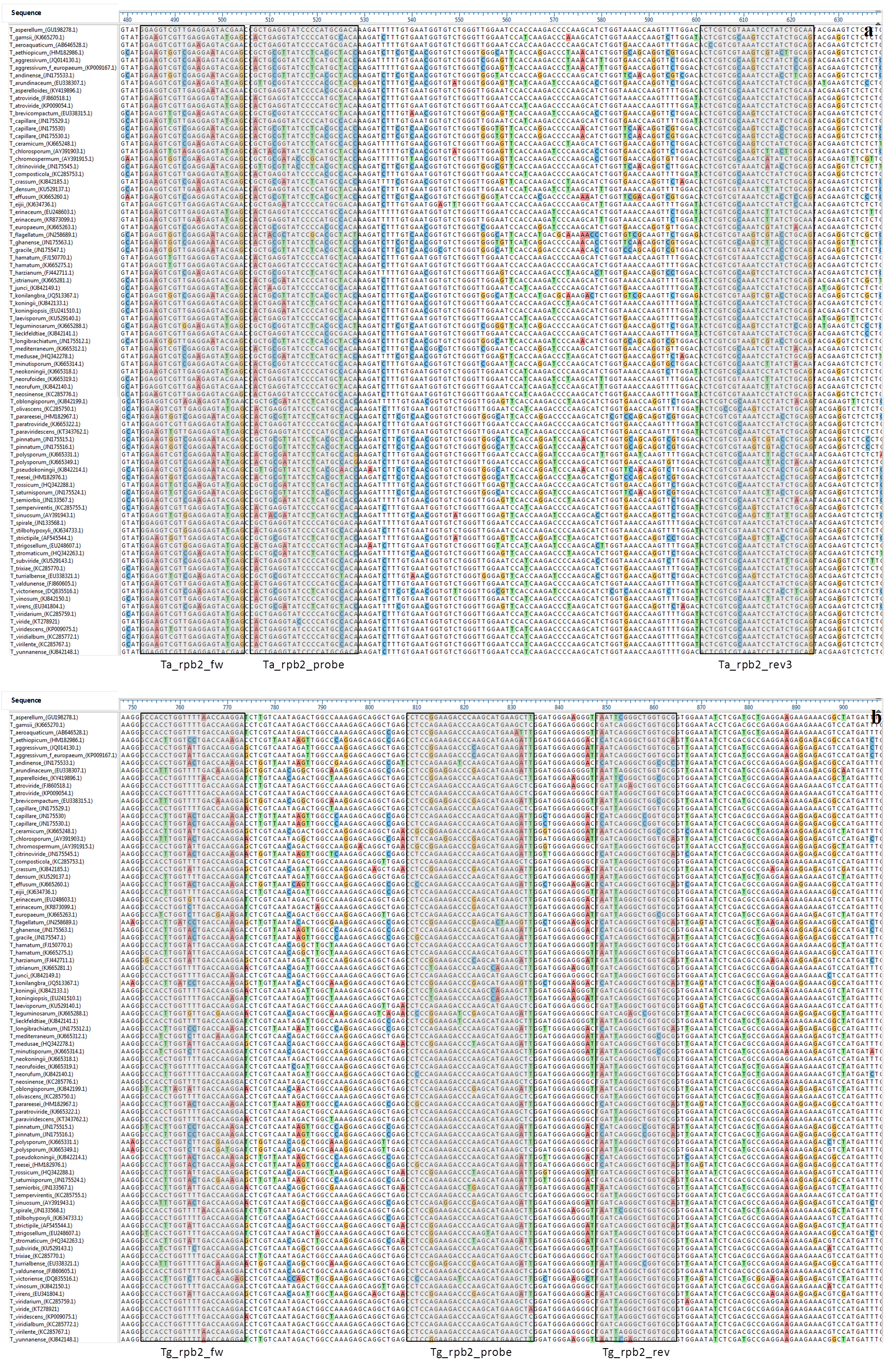

Supplement: FIGURE S1 — SNPs identification in T. asperellum (A) and T. gamsii (B) rpb2 gene sequences for primers/probe sets design. ∗For each Trichoderma spp., GenBank accession number of representative rpb2 gene sequences is included between parenthesis. SNPs as compared to T. asperellum (A) and T. gamsii (B) are displayed with a different color. [file Image_1.TIF]
