# Supplementary material for: A Ready-to-Use Single- and Duplex-TaqMan-qPCR Assay to Detect and Quantify the Biocontrol Agents Trichoderma asperellum and Trichoderma gamsii
Source: Front Microbiol. 2018 Aug 31;9:2073. doi: 10.3389/fmicb.2018.02073 (PMC6127317; doi:10.3389/fmicb.2018.02073)
Supplement: TABLE S1 — Detection of T. asperellum DNA extracted with two extraction protocols by qPCR. ∗50 mg (protocol 1) and 250 mg (protocol 2). n.a., no amplification. Values are mean ± standard error of three biological replicates. [file Table_1.docx]

Supplementary Material

A ready-to-use single- and duplex-TaqMan-qPCR assay to detect and quantify the biocontrol agents *Trichoderma asperellum* and *Trichoderma gamsii*

**D. Gerin, S. Pollastro, C. Raguseo, R.M. De Miccolis Angelini, F. Faretra Correspondence:**

Corresponding Author: stefania.pollastro@uniba.it

**Table S1. Detection of *T. asperellum* DNA extracted through two extraction protocols by qPCR.**

| **Conidia (No.) added to soil*** | **Cq values** | |
| --- | --- | --- |
|  | **Protocol 1** | **Protocol 2** |
| 1×10^7^ | 29.5±0.06 | 23.2±0.23 |
| 1×10^6^ | 34.3±0.40 | 26.8±0.46 |
| 1×10^5^ | 35.4±0.41 | 32.0±0.19 |
| 1×10^4^ | 36.3±0.30 | 34.2±0.13 |
| 1×10^3^ | n.a. | 35.9±0.53 |
| 1×10^2^ | n.a. | n.a. |
| 1×10^1^ | n.a. | n.a. |

* 50 mg (protocol 1) and 250 mg (protocol 2).

n.a.: no amplification.

Values are mean±standard error of three biological replicates.
